# ADC Benchmark Range for Correct Diagnosis of Primary and Recurrent Middle Ear Cholesteatoma

**DOI:** 10.1155/2018/7945482

**Published:** 2018-04-24

**Authors:** Camilla Russo, Andrea Elefante, Antonella M. Di Lullo, Barbara Carotenuto, Alessandra D'Amico, Michele Cavaliere, Maurizio Iengo, Arturo Brunetti

**Affiliations:** ^1^Dipartimento di Scienze Biomediche Avanzate, Università degli Studi di Napoli “Federico II”, Naples, Italy; ^2^Dipartimento di Neuroscienze, Scienze Riproduttive e Odontostomatologiche, Università degli Studi di Napoli “Federico II”, Naples, Italy

## Abstract

**Objectives:**

Magnetic resonance imaging (MRI) and in particular diffusion-weighted imaging (DWI) have been broadly proven to be the reference imaging method to discriminate between cholesteatoma and noncholesteatomatous middle ear lesions, especially when high tissue specificity is required. The aim of this study is to define a range of apparent diffusion coefficient (ADC) values within which the diagnosis of cholesteatoma is almost certain.

**Methods:**

The study was retrospectively conducted on a cohort of 124 patients. All patients underwent first- or second-look surgery because primary or secondary acquired cholesteatoma was clinically suspected; they all had preoperative MRI examination 15 days before surgery, including DWI from which the ADC maps were calculated.

**Results:**

Average ADC value for cholesteatomas was 859,4 × 10^−6^ mm^2^/s (range 1545 × 10^−6^ mm^2^/s; IQR = 362 × 10^−6^ mm^2^/s; *σ* = 276,3 × 10^−6^ mm^2^/s), while for noncholesteatomatous inflammatory lesions, it was 2216,3 × 10^−6^ mm^2^/s (range 1015 × 10^−6^ mm^2^/s; IQR = 372,75 × 10^−6^ mm^2^/s; *σ* = 225,6 × 10^−6^ mm^2^/s). Interobserver agreement with Fleiss' Kappa statistics was 0,96. No overlap between two groups' range of values was found and the difference was statistically significant for *p* < 0.0001.

**Conclusions:**

We propose an interval of ADC values that should represent an appropriate benchmark range for a correct differentiation between cholesteatoma and granulation tissue or fibrosis of noncholesteatomatous inflammatory lesions.

## 1. Introduction

Middle ear cholesteatoma is an enlarging cystic keratin-filled mass surrounded by stratified squamous epithelium often originating from Prussak space or more generally in epitympanum. The most common classification of cholesteatomas is based on pathogenesis, and it differentiates three types of lesion: congenital (2%), primary acquired (80%), and secondary acquired (18%) [1]. Although cholesteatoma is a benignant nonneoplastic lesion, its intrinsic tendency to erode adjacent bony structures and damage anatomical components within the temporal bone makes it locally aggressive and potentially fatal [2]. The diagnosis is mainly performed by physical examination, based on the presence of painless otorrhea, hearing loss, and dizziness; occasionally, first clinical presentation can include symptoms of central nervous system (CNS) complications, potential confounding about the origin of the lesion [3–5].

High resolution computed tomography (HRCT) frequently is the first step in the diagnostic imaging assessment of suspected cholesteatoma because of optimal spatial resolution and high sensitivity for bony erosions; at present, CT is considered a key element in presurgical planning, helpful in depicting precise disease extension [2, 6]. Nevertheless, HRCT is inaccurate for characterizing different types of effusions that can determine middle ear opacification [2, 3]. When high tissue specificity and sensitivity are required, especially when postoperative residual or recurrent disease is suspected [7], it is necessary to resort to magnetic resonance imaging (MRI), already widely used in assessment of ear malformations and hearing loss, temporal bone tumors, or middle ear infections [8–11]. Among different advanced techniques [12], diffusion-weighted imaging (DWI) sequences are the reference method for neuroradiologists to discriminate between middle ear cholesteatoma and noncholesteatomatous lesions [13–18]. On DWI, cholesteatoma is hyperintense in part because of restricted water diffusion, in part because its content of keratin produces high signal intensity in the pathological area (T2-shine-through effect), as shown calculating apparent diffusion coefficient (ADC) values [13]. Among different types of DWI sequences, it has been proved that the best diagnostic accuracy is obtained with non-echo-planar (non-EPI) DWI compared to echo-planar (EPI) techniques [3, 19–21], specifically with multi-shot (Msh) EPI compared to single-shot (Ssh) non-EPI [22], in particular after first-time surgery [23–25]. Therefore, the highest reliability for the detection of middle ear cholesteatoma is at present obtained with MSH-TSE DWI [26]. For diagnostic purposes, it is necessary to confirm the data obtained by calculating ADC values from DWI sequences performed [23]. The aim of this study is to define a range of ADC values within which the diagnosis of cholesteatoma is almost certain.

## 2. Material and Methods

### 2.1. Patients

From April 2011 to March 2016, we recruited 124 consecutive unrelated patients (69 females; 55 males; mean age 35,5 Y) with clinical suspicion of unilateral or bilateral middle ear cholesteatoma, candidate to surgical procedure. We included both primary and secondary acquired suspected lesions. In our sample no congenital cholesteatoma was present. All patients underwent preoperative MRI 15 days before first- or second-look surgery. The diagnosis of cholesteatoma was always confirmed histologically. Overall, 15 patients were unable to complete MRI examination, while 9 additional patients were excluded because of their refusal to undergo surgery. Finally, from the original sample, 100 patients (56 females; 44 males; mean age 34,9 Y) were recruited. Demographic and clinical information of all patients included in the study is shown in Table 1 (Table 1). The protocol was approved by local Ethical Committee and written informed consent was preliminarily obtained from all patients.

### 2.2. Imaging Technique and Evaluation

**Figure 1:**
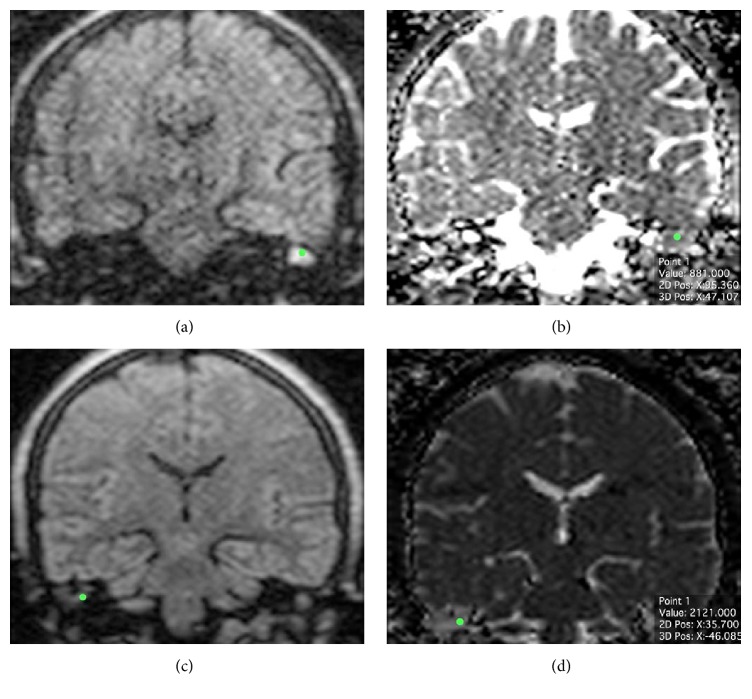
((a)-(b)) MSH-TSE DWI of a group 1 patient (ch+), with the calculated ADC value on ADC cartography (881 × 10^−6^ mm^2^/s); (c)-(d) MSH-TSE DWI of a group 2 patient (ch−), with the calculated ADC value on ADC cartography (2121 × 10^−6^ mm^2^/s).

MRI was performed at 1.5 T MR unit (Philips Intera, Philips Medical Systems, Netherlands) with an 8-channel head coil. The imaging protocol consisted in TSE T2w on the axial plane (11 slides; TR 3000 ms; TE 120 ms; thickness 3.00 mm; FA 90; phase I > S; view size 2338 × 1228; matrix 288 × 288; 6 averages); T1w SE on three orthogonal axes before and after intravenous administration of contrast media (11 slides/plane; TR 550 ms; TE 15 ms; thickness 3.00 mm; FA 90; view size 2338 × 1228; matrix 256 × 256; 3 averages); T2 3D DRIVE on the axial plane for the study of inner ear and cerebellopontine angle (50 slides; TR 1500 ms; TE 250 ms; thickness 1.40 mm; FA 90; phase I > S; view size 2338 × 1228; matrix 320 × 320; 6 averages); coronal multi-shot non-echo-planar diffusion-weighted imaging (MSH non-EPI DWI). DWI acquisition was performed on the coronal plane (20 slides; TR 3000 ms; TE 82,44 ms; thickness 3.00 mm; FA 90; phase R > L; view size 2338 × 1228; matrix 128 × 128; *b* = 0 and *b* = 800 s/mm^2^; 5 averages); during Msh-TSE DWI, cardiac gating was performed in order to limit patient-related artefacts due to heart pulse and blood flow. ADC maps were obtained by using the free Osirix plugin “ADC Map Calculation.” For each patient, three different neuroradiologists measured ADC values separately. A circular region of interest (ROI) of 1 mm in diameter was placed in the area where abnormal restricted diffusion was more evident on DWI sequence with the highest *b*-values (*b* = 800) by every single observer and then automatically transferred to the coregistered ADC map, as shown in Figure 1 (Figure 1); the average value of ADC within the ROI was considered.

### 2.3. Statistical Analysis

For final analysis, we considered the arithmetic mean of measured ADC values obtained by the three neuroradiologists. Considering the normal distribution of the collected data assessed by using the Shapiro-Wilk Test for normality, parametric statistical analysis was performed with Student's *t*-test (MEDCALC® Statistical Software). We compared the average ADC values of histologically confirmed cases of cholesteatoma with the ones of histologically identified noncholesteatomatous inflammatory lesions.

## 3. Results

**Figure 2:**
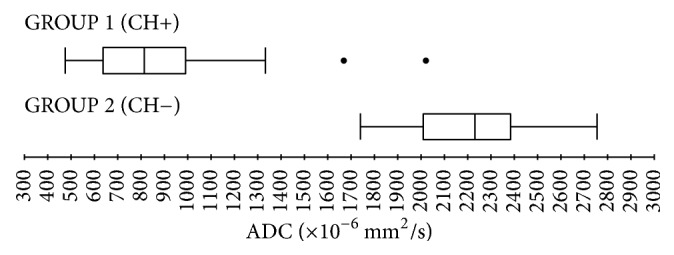
Boxplots representing ADC values distribution in group 1 (patients with histologically confirmed cholesteatoma) and group 2 (histologically identified noncholesteatomatous inflammatory lesions).

In 72 cases (Group 1; ch+) primary or secondary acquired cholesteatoma was confirmed histologically, while in 28 cases (Group 2; ch−) postoperative findings indicated the presence of noncholesteatomatous inflammatory tissue. Overall agreement among raters calculated using Fleiss' Kappa statistics was 0,96 (Free Marginal Kappa = 0,93). Average ADC value for cholesteatomas was 859,4 × 10^−6^ mm^2^/s (lowest value *Q*_0_ = 477 × 10^−6^ mm^2^/s; highest value *Q*_4_ = 2022 × 10^−6^ mm^2^/s; range 1545 × 10^−6^ mm^2^/s; IQR = 362 × 10^−6^ mm^2^/s; standard deviation *σ* = 276,3 × 10^−6^ mm^2^/s). Average ADC value for noncholesteatomatous inflammatory lesions was 2216,3 × 10^−6^ mm^2^/s (lowest value *Q*_0_ = 1741 × 10^−6^ mm^2^/s; highest value *Q*_4_ = 2756 × 10^−6^ mm^2^/s; range 1015 × 10^−6^ mm^2^/s; IQR = 372,75 × 10^−6^ mm^2^/s; standard deviation *σ* = 225,6 × 10^−6^ mm^2^/s). No overlapping value between the two groups was found; two outlier values (below *Q*_1_ − 1.5 × IQR or above *Q*_3_ + 1.5 × IQR) were identified, both in the group of patient with histologically confirmed cholesteatoma and candidate to second-look surgery (Figure 2). The difference between the two groups was statistically significant for *p* < 0.0001. No significant difference was found between ADC values in primary and secondary cholesteatoma.

## 4. Discussion

To our knowledge, this report is the first quantitative assessment of a specific range of ADC values for differentiating between middle ear cholesteatoma and noncholesteatomatous tissue on a relatively large number of patients.

Reproducibility and reliability of ADC values in the head and neck region of healthy subjects have been recently assessed, and the importance of MR imaging systems and sequences performed has been pointed out [27]. With this knowledge, we tried to systematically investigate accuracy and reliability of ADC range values in determining whether middle ear lesions should be attributed to cholesteatoma with a 1.5 T magnetic resonance field, widely used in clinical practice and appropriate to diagnostic purposes, as previously demonstrated [28].

Previous consensus conferences proposed standardized criteria for ROI placement in diagnosis and response assessment of expansive and tumoral lesions [29], suggesting that tumor ROI definition should be done on traditional high-contrast images (i.e., T2-weighted) and then transferred to the DWI data set. Considering the nonneoplastic nature of cholesteatoma, in this study, we opted for a small hand-drawn ROI as commonly used in clinical daily practice. The choice of ROI size (circular, 1 mm in diameter), even if potentially prone to errors, is prompted by the effort to identify even small cholesteatomas (≈3 mm in diameter). The rationale behind this option is to exclude from the evaluation tissue and/or anatomical structures surrounding the suspected cholesteatoma. Indeed, even if not representing the whole lesion, a very small ROI may help to discriminate the portion of residual or recurrent cholesteatoma from granulation and inflammatory tissue all around; this is particularly important in case of large recurrent lesions with small inner areas of restricted water diffusion on DWI.

As the hyperintensity of cholesteatoma on DWI is a combination of T2 shine-through effect and restricted water diffusion, to confirm the diagnostic suspicion it is always desirable to compare the DWI image to the ADC maps [23]. In case of true restricted diffusion, as happens with cholesteatoma, the area of increased DWI intensity will coincide with the low signal area on ADC map [13, 30]. This appearance on DWI and on relative ADC map makes it possible to distinguish cholesteatoma from noncholesteatomatous inflammatory lesions, especially in case of doubt before second-look surgery [1, 24, 25, 31]. In addition to these considerations, it is widely recognized that quantitative ADC measurements are able to improve specificity compared to the DWI subjective qualitative assessment [32]. Indeed, ADC helps to identify false positive cases in which the misdiagnosis of cholesteatoma is due to the presence of confounding factors, including cerumen, haemorrhage following recent surgery, middle ear packing materials, autologous and heterologous bone replacement materials, or infections [3, 19, 23, 33].

In the light of previously described results on smaller samples of patients [13, 30, 32, 34], we found two different groups of values not overlapping one another: patients with cholesteatoma were found to have lower ADC values (Group 1; ch+ median 822 × 10^−6^ mm^2^/s) compared to patients without cholesteatoma (Group 2; ch− median 2233 × 10^−6^ mm^2^/s) (Figure 2). The difference in intensity signal is mainly due to the significant amount of granulation tissue and/or fibrosis of noncholesteatomatous inflammatory lesions. Two outlier values (below *Q*_1_ − 1.5 × IQR or above *Q*_3_ + 1.5 × IQR) were identified in the group of patient candidate to second-look surgery and with histologically confirmed cholesteatoma. In both cases, these findings are probably attributable to a small residual/recurrent cholesteatoma surrounded by a large amount of granulation tissue. In fact, DWI and ADC ability to detect small cholesteatomas is limited to lesions bigger than 2-3 mm [35]; therefore, small residual/recurrent pearls within granulation tissue resulting from first-time surgery can be easily missed.

**Figure 3:**
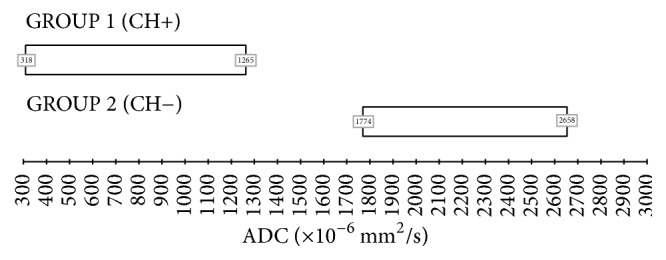
Proposed ADC ranges (mean ADC value ± 1,96 standard deviation *σ* → confidence interval 95%). (1) Reference range for cholesteatoma: 318 × 10^−6^ mm^2^/s–1265 × 10^−6^ mm^2^/s. (2) Reference range for noncholesteatomatous inflammatory lesions: 1774 × 10^−6^ mm^2^/s–2658 × 10^−6^ mm^2^/s.

Given the sufficiently large sample of this study and the distribution of the collected data, we propose a benchmark interval of ADC for both cholesteatoma and noncholesteatomatous inflammatory lesions ranging from ADC ±1,96 standard deviation *σ* (confidence interval 95%). For cholesteatoma, after excluding the two above-mentioned outliers, we suggest a reference range between 318 × 10^−6^ mm^2^/s and 1265 × 10^−6^ mm^2^/s, while for noncholesteatomatous inflammatory lesions we suggest a reference range between 1774 × 10^−6^ mm^2^/s and 2658 × 10^−6^ mm^2^/s (Figure 3).

No significant difference was found stratifying patients by primary and secondary acquired cholesteatoma or by age and sex. For second-look surgery, neither autologous nor heterologous materials have been used in middle ear surgical repair in our sample, in order to prevent scan artefacts that could potentially affect the interpretation of the ADC map in secondary lesions. A further core strength of this work is the large number of highly selected patients recruited over the years compared to the small sample size of previous studies [30, 32, 34], which did not allow to generalize the obtained results and to identify specific ranges of values. An extensive comparison between studies regarding ADC values in suspected middle ear cholesteatoma assessment is presented in Supplementary Table 1. Homogeneous sample characteristics can be considered both a strength but also a possible detriment, because we did not include any other kind of lesion with potentially overlapping ADC values. In fact, on the other hand, the main limitation is the absence of middle ear abscesses, whose ADC values should have represented an additional range to the ones described in the study [34]. Moreover, our results related to the MR unit and the specific DWI sequences used, so their validity in different conditions should be confirmed by further studies, being ADC values reproducibility potentially influenced by coil system, specific sequence performed, and field strength [27]. Lastly, subjective placement of a small ROI within the lesions on higher *b*-values DW images can be prone to error because of T2-shine-through effect, occasionally affecting the goodness of final result.

## 5. Conclusions

In conclusion, ADC maps could be helpful in differentiating cholesteatoma from noncholesteatomatous inflammatory lesions, in particular when postoperative residual or recurrent disease is suspected and differential diagnosis is difficult to perform. We suggest a reference range between 318 × 10^−6^ mm^2^/s and 1265 × 10^−6^ mm^2^/s for cholesteatoma and between 1774 × 10^−6^ mm^2^/s and 2658 × 10^−6^ mm^2^/s for noncholesteatomatous lesions. The proposed interval of ADC values should represent an appropriate benchmark range for a correct and unambiguous interpretation of what is observed in non-EPI DWI sequences.

## Figures and Tables

**Table 1:** Demographical and surgical information of patients included in the MRI analysis.

	Primary cholesteatoma (*N* = 54)	Secondary cholesteatoma (*N* = 46)
Age (mean ± SD)	35.43 ± 17.97	36.86 ± 18.35
Sex (M/F)	24/30	10/36
Side (DX/SN)	37/17	16/30
Histology (+/−)	42/12	28/18

## Abbreviations

MRI: Magnetic resonance imaging
HRCT: High resolution computed tomography
CNS: Central nervous system
DPI: Delayed postcontrast T1-weighted MR imaging
DWI: Diffusion-weighted imaging
ADC: Apparent diffusion coefficient
EPI: Echo-planar imaging
MSH-TSE: Multi-shot turbo spin-echo
Ssh EPI: Single-shot echo-planar
ROI: Region of interest
IQR: Interquartile range.

## Supplementary Materials

Supplementary Materials In supplementary material, a table comparing DWI and ADC maps data of all studies concerning cholesteatoma versus noncholesteatomatous tissue is reported. Supplementary Table 1: table comparing studies reporting cholesteatoma versus noncholesteatoma DWI and ADC values.
