# Supplementary material for: ADC Benchmark Range for Correct Diagnosis of Primary and Recurrent Middle Ear Cholesteatoma
Source: Biomed Res Int. 2018 Apr 24;2018:7945482. doi: 10.1155/2018/7945482 (PMC5941773; doi:10.1155/2018/7945482)
Supplement: Supplementary Materials — In supplementary material, a table comparing DWI and ADC maps data of all studies concerning cholesteatoma versus noncholesteatomatous tissue is reported. Supplementary Table 1: table comparing studies reporting cholesteatoma versus noncholesteatoma DWI and ADC values. [file 7945482.f1.pdf]

SUPPLEMENTARY MATERIAL

Supplementary Table 1. *Table comparing studies reporting cholesteatoma vs non-cholesteatoma DWI and ADC values.*

Authors and Year	N	MRI (T)	Type of CH	ROI	Considered Variable	Range CH+	Mean Value CH+	Range CH-	Mean Value CH-	IOA
Russo et al. 2017	100	1.5	Primary + Recurrent vs Granulation	Circular (∅=1mm)	ADC (UM=mm2/s)	318×10^{-6} - 1265×10^{-6}	$859,4\times10^{-6}$	1774×10^{-6} - 2658×10^{-6}	$2216,3\times10^{-6}$	0.96
Suzuki et al. 2014	29	3	Primary vs Recurrent vs Granulation	Freehand	SIR	1,42 – 5,18; 1,22 – 5,99	3,75; 3,28	1,42 – 3,58	2,38	NA
Lingam et al. 2013	56	1.5	Primary + Recurrent vs Granulation	Freehand	ADC (UM=mm2/s)	539×10^{-6} - 858×10^{-6}	$707\ \times10^{-6}$	1574×10^{-6} - 1982×10^{-6}	1849×10^{-6}	0.97
Thiriat et al. 2009	15	1.5	CH vs Abscess	Freehand (A≈11mm^2)	ADC (UM=mm2/s)	628×10^{-6} - 1054×10^{-6}	903×10^{-6}	107×10^{-6} - 568×10^{-6}	415×10^{-6}	NA
Vercruysse et al. 2006	20	1.5	Primary	NA	ADC (UM=mm2/s)	$664x10^{-6}$ - $1107x10^{-6}$	$844x10^{-6}$	NA	NA	NA

Legend: N = Number of patients included in the study; MRI = Magnetic Resonance Imaging; T = Tesla (field strength); ROI = Region Of Interest; CH = Cholesteatoma; IOA = Inter-Observer Agreement; ADC = Apparent Diffusion Coefficient; ∅ = diameter; UM = Unit of Measurement; SIR = Signal/Intensity Ratio; NA = Not Available; A = Area.
